# Biological Evaluation and In Silico Study of Benzoic Acid Derivatives from *Bjerkandera adusta* Targeting Proteostasis Network Modules

**DOI:** 10.3390/molecules25030666

**Published:** 2020-02-04

**Authors:** Katerina Georgousaki, Nikolaos Tsafantakis, Sentiljana Gumeni, George Lambrinidis, Victor González-Menéndez, Jose R. Tormo, Olga Genilloud, Ioannis P. Trougakos, Nikolas Fokialakis

**Affiliations:** 1 Division of Pharmacognosy and Natural Products Chemistry, Department of Pharmacy, National and Kapodistrian University of Athens, 157 71 Athens, Greece; kat_georgousaki@hotmail.com (K.G.); ntsafantakis@pharm.uoa.gr (N.T.); 2 Department of Cell Biology and Biophysics, Faculty of Biology, National and Kapodistrian University of Athens, 157 72 Athens, Greece; sgumeni@biol.uoa.gr (S.G.); itrougakos@biol.uoa.gr (I.P.T.); 3 Division of Pharmaceutical Chemistry, Department of Pharmacy, National and Kapodistrian University of Athens, 157 84 Athens, Greece; lamprinidis@pharm.uoa.gr; 4 Fundacion MEDINA, Health Sciences Technology Park, 18016 Granada, Spain; victor.gonzalez@medinaandalucia.es (V.G.-M.); ruben.tormo@medinaandalucia.es (J.R.T.); olga.genilloud@medinaandalucia.es (O.G.)

**Keywords:** fungi, *Bjerkandera adusta*, benzoic acid derivatives, cathepsins activity, molecular docking, proteasome activity

## Abstract

A main cellular functional module that becomes dysfunctional during aging is the proteostasis network. In the present study, we show that benzoic acid derivatives isolated from *Bjerkandera adusta* promote the activity of the two main protein degradation systems, namely the ubiquitin-proteasome (UPP) and especially the autophagy-lysosome pathway (ALP) in human foreskin fibroblasts. Our findings were further supported by in silico studies, where all compounds were found to be putative binders of both cathepsins B and L. Among them, compound **3** (3-chloro-4-methoxybenzoic acid) showed the most potent interaction with both enzymes, which justifies the strong activation of cathepsins B and L (467.3 ± 3.9%) on cell-based assays. Considering that the activity of both the UPP and ALP pathways decreases with aging, our results suggest that the hydroxybenzoic acid scaffold could be considered as a promising candidate for the development of novel modulators of the proteostasis network, and likely of anti-aging agents.

## 1. Introduction

The 26S proteasome and lysosomal cathepsins activities are affected permanently or transiently by aging or exposure to environmental stress [1,2,3]. The ubiquitin-proteasome pathway (UPP) is the primary protein degradation pathway of the cell and a main part of the proteostasis (proteome stability) network (PN), which controls protein synthesis, folding, trafficking and degradation [3,4,5]. The proteasome holoenzyme comprises of the 20S core particle which binds to the 19S regulatory particles to form the 26S proteasome. The 20S proteasome is a barrel-like structure of four stacked heptameric rings (α- and β-type) with the caspase- (C-L; LLE), trypsin- (T-L; LRR), and chymotrypsin- (CT-L; LLVY) like enzymatic activities located at the β1, β2, and β5 subunits, respectively. The 19S RP is involved in substrate recognition, de-ubiquitination, unfolding and translocation into the 20S cylinder [6]. In contrast to UPP that targets mostly short-lived proteins, the autophagy-lysosome pathway (ALP) degrades cytoplasmic portions, long-lived proteins, as well as entire cellular organelles. Protein aggregates or large inclusions can be directed to the lysosome, a membrane-bound organelle containing several nonspecific proteases (e.g., cathepsins; especially B, L and D) which can degrade a wide range of substrates [7].

*Bjerkandera adusta,* commonly known as smoky polypore, is a wood-rotting basidiomycete well known for its high ligninase activity [8]. It belongs to the white-rot fungi, who are the most efficient lignin degraders in nature [9,10,11]. Studies have highlighted the production of halogenated organic compounds by *B. adusta* [12,13], which not only play an important role in lignin degradation but they also have an antibiotic effect [14,15]. Chlorinated anisyl metabolites (CAM) and derivatives are the most common organohalogens produced by basidiomycetes and in particular by species of the genus *Bjerkandera* [12,16,17,18].

Natural compounds are recognized for their broad range of biological activities with numerous applications in health and disease treatment [19,20,21]. In continuation of our studies in microbial derived bioactive metabolites, we investigated the strain *B. adusta* CF-092983 as a potential source of natural products that are able to enhance UPP and ALP functionalities and thus to contribute to the maintenance of proteome stability affected by several exogenous and/or endogenous factors [22].

## 2. Results and Discussion

### 2.1. Extraction and Bioevaluation

As the maintenance of proteostasis is considered a promising target for discovering small active molecules with anti-aging activity from microorganisms, *B. adusta* (CF-092983) was selected for further investigation [21]. The lyophilized biomass of 1 L culture of the fungal strain was successively extracted using different organic solvents of increasing polarity: n-Hex, EtOAc, and 1:1 MeOH/H_2_O. After the evaporation under reduced pressure of the methanolic part of the hydroalcoholic extract, a liquid/liquid partition with EtOAc (EtOAc-LL) was followed. The remaining aqueous part was treated with XAD-4 resin and eluted with MeOH. All extracts were then forwarded for HPLC analysis, where both EtOAc extracts showed the richest secondary metabolites content (see Supplementary Materials, Figure S1). Thus, they were chosen for further biological and chemical investigation.

**Figure 1:**
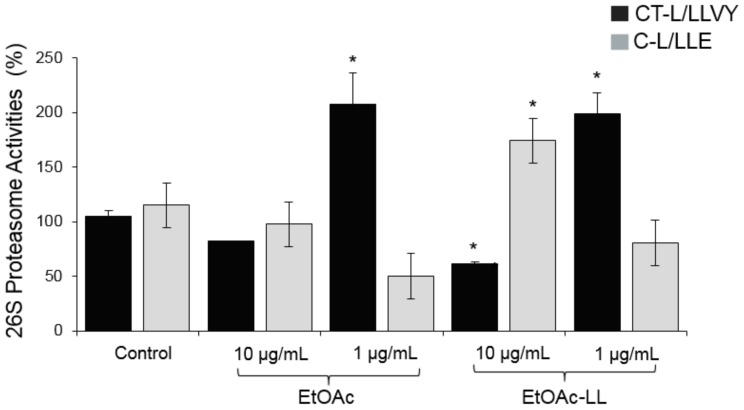
Relative (%) 26S proteasome activities of CT-L (LLVY/β5) and C-L (LLE/β1) in normal replicating (young) human fibroblasts after 24 h of treatment with the EtOAc and EtOAc-LL extracts of the strain CF-092983 at concentrations of 10 μg/mL and 1 μg/mL. Bars, ± SD (*n* = 3). * *p* < 0.05. Controls (cells treated with the vehicle/DMSO) were set to 100%.

**Figure 2:**
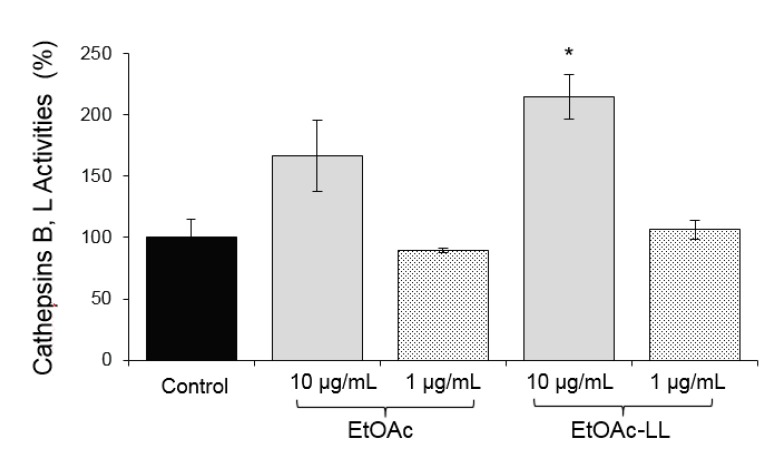
Relative (%) cathepsins B, L activities in normal replicating (young) human fibroblasts after 24 h of treatment with the EtOAc and EtOAc-LL extracts of the strain CF-092983 at concentrations of 10 μg/mL and 1 μg/mL. Bars, ± SD (*n* = 3). * *p* < 0.05. Controls (cells treated with the vehicle/DMSO) were set to 100%.

Evaluation of proteasome and cathepsin activities of the generated EtOAc extracts was performed on normal replicating (young) human foreskin fibroblasts in two different concentrations (1 and 10 μg/mL). The most promising overall activity was exhibited by the EtOAc-LL extract at 10 μg/mL, since it combined the trend to enhance, at least, one of the main activities of the proteasome (C-L/LLE at 10 μg/mL and CT-L/LLVY at 1 μg/mL) and of cathepsins B and L (Figure 1 and Figure 2); at these concentrations no cytotoxicity was noted in human foreskin fibroblasts (data not shown).

Further evaluation of the cytotoxicity of the EtOAc-LL extract following various cell growth inhibition assays were carried out in two different cancer cell lines (Hep-G2 and A2058), as well as in normal skin fibroblasts (CCD25sk). Our results showed no cytotoxicity in Hep-G2, A2058 and CCD25sk cell lines (4.81 ± 0.28%, 5.02 ± 0.18%, 3.56 ± 4.06%, of cell growth inhibition respectively). Therefore, considering the inducing effects of the EtOAc-LL extract on proteasome and lysosome activities, we focused on this extract as the most promising for further chemical investigation.

### 2.2. Isolation

**Figure 3:**
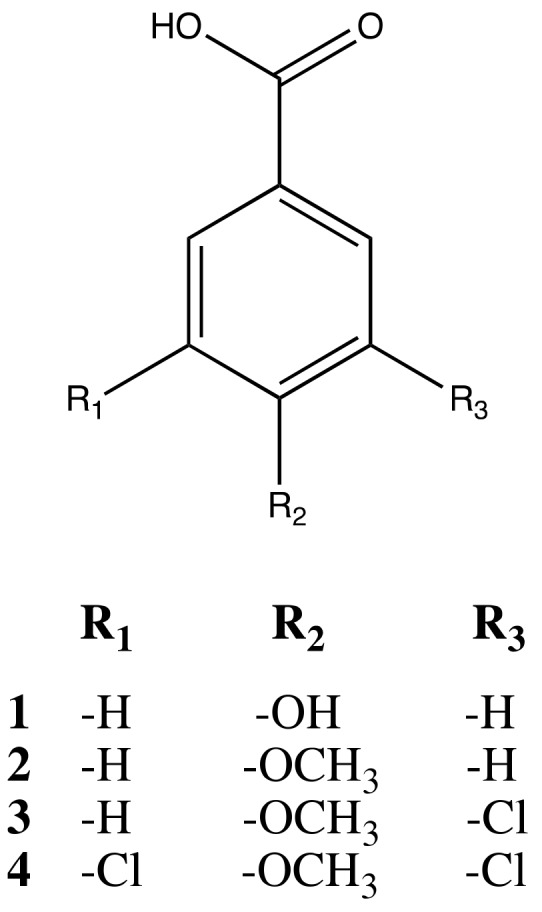
Chemical structures of the isolated compounds **1**-**4**.

Comparative HPLC analysis of the extracts showed the presence of four main compounds, (Figure S1) mainly distributed in the EtOAc-LL extract. Thus, it was hypothesized that the bioactivity of the EtOAc-LL was attributed to the presence of those compounds. A purification process using semi-preparative HPLC was followed and the structures of compounds **1**–**4** (Figure 3) were elucidated by the extensive use of 1D and 2D-NMR spectroscopy, supported by HRMS spectra and by comparison with literature data (Figures S2–S11) [23,24,25,26].

Compound **1** was characterized as a 4-hydroxy derivative of benzoic acid while compound **2** as its 4-methoxy derivative. The downfield shift observed for the equivalent methine groups C-2 and C-6 at 7.98 ppm and 129.8 ppm of compound **4** in combination to the uperfield shift of the quaternary carbons C-3 and C-5 at 129.4 ppm suggested a di-halogenated molecule. Similarly, the characteristic chemical shifts and multiplicity of the ^1^H-NMR spectra of compound **3** suggested the presence of a mono-halogenated benzoic acid derivate. Hypothesized structures of compounds **1**–**4** were confirmed by UPLC-HRMS spectra data analysis. The characteristic isotope pattern at *m*/*z* 185 and *m*/*z* 187 ([M − H]^−^ and [M + 2 − H]^−^) for compound **3** (Figure S7) and at *m*/*z* 218, 220, 222 ([M − H]^−^, [M + 2 − H]^−^, [M + 4 − H]^−^) for compound **4** (Figure S11) confirmed the presence of one and two chlorine atoms, respectively.

Compound **1** was detected as the most abundant benzoic acid derivative. A HPLC method was employed to quantify its content in the active EtOAc and EtOAc-LL extracts. The linear regression has shown good linearity in the investigated ranges (*r*^2^ = 0.9952) and the amount of this compound in the EtOAc LL was 17.57 ± 0.18 μg/mg of extract, an increment of 506.2% with respect to the amount in the EtOAc extract. Similar to compound **1**, compounds **2**, **3** and **4** shown to be of higher abundance in the EtOAc LL extract (Table 1).

### 2.3. Biological Evaluation of Isolated Compounds

**Figure 4:**
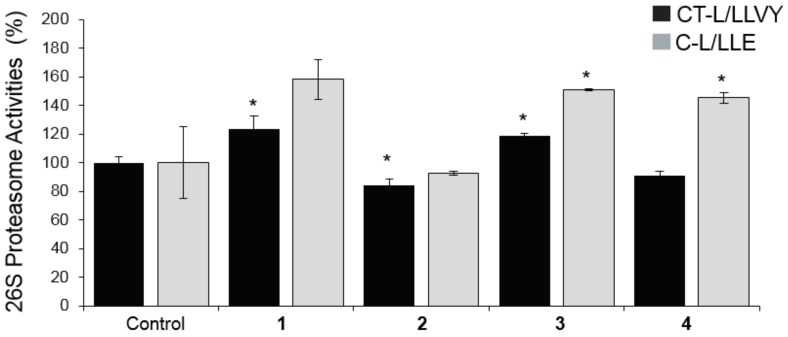
Relative (%) 26S proteasome activities of CT-L (LLVY/ β5) and C-L (LLE/ β1) in normal replicating (young) human fibroblasts after 24h of treatment with the compounds **1**, **2**, **3** and **4** at a concentration of 5 μΜ. Bars, ± SD (*n* = 3). * *p* < 0.05. Controls (cells treated with the vehicle/DMSO) were set to 100%.

**Figure 5:**
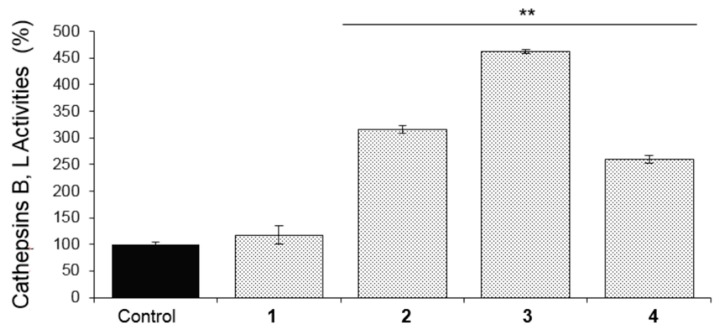
Relative (%) cathepsins B and L activities in normal replicating (young) human fibroblasts after 24 h of treatment with the compounds **1**, **2**, **3** and **4** at a concentration of 5 μΜ. Bars, ± SD (*n* = 3). ** *p* < 0.01. Controls (cells treated with the vehicle/DMSO) were set to 100%.

As our findings showed an enhanced bioactivity for the EtOAc-LL extract (Figure 1 and Figure 2) and a higher content of compounds **1**, **2**, **3** and **4**, a detailed biological evaluation in cell-based assays of the aforementioned compounds was followed to assign the activity of each metabolite. With the exception of compound **2**, the proteasomal chymotrypsin-like (CT-L/LLVY) and caspase like (C-L/LLE) activities were induced by compounds **1**, **3**, and **4**, at a concentration of 5 μM (Figure 4); among them, compound **1** and **3** showed the greatest potential (Figure 4). In all cases, a higher activation of the rate-limiting for protein breakdown chymotrypsin like enzymatic activity (CT-L; LLVY) was observed. Regarding cathepsins B, L activation, compound **3**, which is characterized as a mono-halogenated benzoic acid derivate, showed the greatest bioactivity (Figure 5), while compound **1** showed no significant activity (Figure 5). Our results suggest that the effects of *B. adusta* compounds on autophagy-lysosome pathway are likely more potent than that on proteasome. To our knowledge, this is the first report describing the influence of benzoic acid derivatives on proteostasis network modules (UPP, ALP) functionality.

Compounds **1** (4-hydroxybenzoic acid) and **2** (4-methoxybenzoic acid) have been previously isolated from different bacterial [27,28,29,30] and fungal strains [31,32,33,34,35]. However, they have never been reported from a fungus strain belonging to the genus of *Bjerkandera*. On the other hand, compounds **3** (3-chloro-4-methoxy benzoic acid) and **4** (3,5-dichloro-4-methoxybenzoic acid) have previously been reported from *Bjerkandera* sp. BOS55 and *B. adusta* BEUK47 [16]. Moreover, both compounds **3** and **4** have been detected in a soil sample associated with the mushroom *Lepista nuda* [36], while compound **4** has also been isolated from the basidiomycete *Stropharia squamosa* and from a fungus belonging to the genus *Penicillium* [24,37].

Several studies have investigated the antibiotic [38,39], antifungal [40], antiproliferative [41], tyrosinase inhibitory activity [42], antioxidant [43], antidiabetic [44] and cytotoxic effects [45,46] of compound **1** and **2**. Regarding compound **4**, it has been found to inhibit neurolysin and the angiotensin-converting enzyme [47] while no bioactivity has been reported yet for compound **3**. It is worth mentioning that, during our investigation, compound **3** showed the highest activation of cathepsins B, L and together with compound **1** the stronger inducing effect on proteasome activity.

### 2.4. Molecular Docking Simulation of the Binding Mode of the Isolated Compounds with Procathepsins B and L

Cathepsin activation is achieved by a proteolytic cleavage of a specific part of the proenzyme named procathepsin. This process is followed by conformational rearrangement of the mature enzyme [48]. Two major mechanisms are known, the autocatalysis and trans-activation. Autocatalysis, starts in low pH environment where N-terminal prodomain is cleaved off. The mature cathepsin initiate a chain reaction to facilitate the cleavage of the N-terminal peptide of other procathepsin enzyme [49]. Glycosaminoglycans (GAGs) are found to modulate the procathepsin activation. They bind between the occluding loop and the prodomain via electrostatic interactions, and they induce conformational changes to unmask the active site facilitating the access to procathepsin molecule. Even short oligosaccharides (4-mer GAGs) are able to activate procathepsins [50]. Thus, our hypothesis is that compounds **2**, **3** and **4** by possessing acidic and bulky groups such as the methoxy or/and chlorium atoms, facilitate the N-terminus rearrangement upon binding.

**Figure 6:**
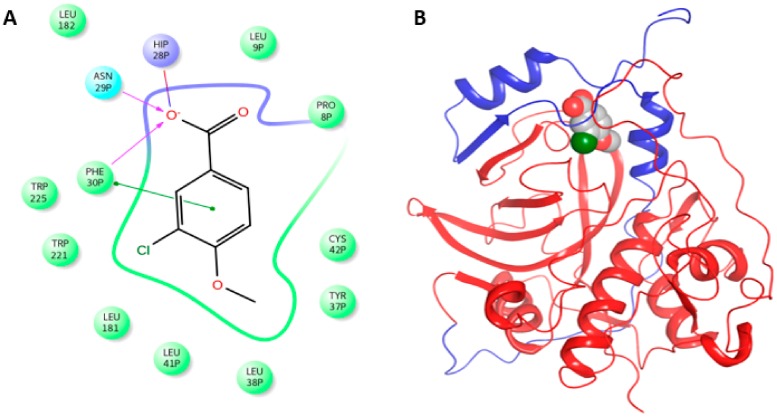
Ligand interaction diagram of global minimum structure of compound **3** in complex with procathepsin B (**A**). Ribbon representation of procathepsin B in complex with global minimum structure of compound **3**. Prodomain is colored with blue ribbons and cathepsin is colored with red ribbons (**B**).

All compounds were bound to both enzymes with Glide Score −7.6 to −9.18 Kcal/mol for procathepsin B and −5.30 to −6.41 Kcal/mol for procathepsin L respectively. Compound **3** showed the most strong interaction with the enzymes as demonstrated in Figure 6 by the interaction of compound **3** with procathepsin B. The carboxyl group of compound **3** forms a salt bridge with HisP^28^ and a hydrogen bond with AsnP^29^ and PheP^30^, while the rest of the molecule is buried to a hydrophobic pocket between cathepsin and the prodomain. Moreover, the benzyl ring is parallel with PheP^30^ interacting with π-π interaction (Figure 6); a similar binding mode and interactions were found for procathepsin L.

## 3. Materials and Methods

### 3.1. Collection and Characterization of the Strain CF-092983

The fruiting body of the fungus (CF-092983) was collected in Navaluenga, Avila, Spain and it was used for the isolation of an axenic culture as previously described [51]. Frozen stock cultures in 10% glycerol (−80 °C) are maintained in the culture collection of Fundación MEDINA. DNA extraction, PCR amplification and DNA sequencing were performed following an already described process [52]. The initial identification of the strain was based on the morphological characteristics of the fruiting body, and confirmed by ITS1-5.8S-28S sequence comparison with sequences from GenBank and NITE Biological Resource Centre using the BLAST application. Sequence homology searches of the strain complete sequence in these databases showed 99–100% of homology with several *B. adusta* strains such as VKM: F-4751, X-41 and X-23, confirming the initial taxonomical identification.

### 3.2. Fermentation and Extraction

*B. adusta* (CF-092983) was fermented by inoculating ten mycelium agar plugs into one 250 mL Erlenmeyer flask containing 50 mL of SMYA medium (bacto neopeptone 10 g, maltose 40 g, yeast extract 10 g, agar 3 g, and H_2_O 1 L). The flask was incubated in a shaking incubator at 220 rpm, at 22 °C and 70% relative humidity. After 7 days of incubation, a 3 mL aliquot of the inoculum was used to inoculate ten flasks of the production medium Dex-Soy [22]. The flasks (100 mL medium per 500 mL Erlenmeyer flask) were incubated in a shaking Khüner incubator at 220 rpm, 22 °C and 70% of relative humidity for 14 days. The scaled-up fermentation broth (1 L) was then homogenized, mixed with acetone (1 L) under continuous shaking at 220 rpm for 1 h and concentrated to 1 L under a stream of nitrogen, before being forwarded to a freeze-drying process. A 30 min ultrasound-assisted extraction was applied to the lyophilized material, using n-Hex (2×), EtOAc (2×), and 1:1 MeOH/H_2_O (2×) consequently. The MeOH was evaporated in a Rotavapor Buchi R-120 (BUCHI Labortechnik AG, Flawil, Switzerland), and the remaining extract was forwarded to liquid-liquid extraction with EtOAc (3 times), which was also concentrated until dryness. The remaining aqueous part was treated with XAD-4 and eluted with MeOH.

### 3.3. Isolation of Compounds

Semi-preparative HPLC was performed using a ThermoFisher Scientific system (San Jose, CA, USA) consisting of a SpectraSystem P4000 pump coupled with a degasser SpectraSystem 1000, a SpectraSystem AS3000 autosampler and an UV SpectraSystem UV6000LP. The system was controlled using ChromQuest 4.1 Chromatography Software. The purification of compounds **1** (RT = 21.2 min, 7.9 mg), **2** (RT = 31.8 min, 1.5 mg), **3** (RT = 37.9 min, 4.1 mg) and **4** (RT = 49.1 min, 1.8 mg) was performed on a Fortis C18 (250 cm × 10 mm, 5 μm) semi-preparative column at the flowrate of 2 mL/min using a mobile phase consisting of H_2_O, 0.1% TFA (Solvent A) and CH_3_CN (Solvent B). The gradient was 0 min/5% B, 20 min/43% B, 30 min/43% B, 60 min/100% B, 65 min/100% B, 67 min/5% B, 70 min/5% B. Injection volume was set at 20 μL and samples were prepared at the concentration of 7 mg/mL using MeOH (HPLC grade). Collection wavelengths were set at 254, 287 and 310 nm.

### 3.4. HPLC Profiling of the Extracts and Quantification of Compound 1

The chemical profile of the extracts, as well as the quantification of **1** were performed on an Agilent 1100 HPLC System (Santa Clara, CA, USA) using a XBridge C18 column (4.6 × 150 mm, 3.5 μm). Solvent A was H_2_O (0.1% TFA) and solvent B MeOH. The gradient was 0 min/10% B, 15 min/45% B, 30 min/100% B, 35 min/100% B, 37 min/10% B, 43 min/10% B, using a flow rate of 1 mL/min. The injection volume was set at 20 μL (extracts tested at concentration of 1 mg/mL) and the UV signal was recorded at 210 and 254 nm. The same method and conditions were used also for the standard curve of compound **1** (RT = 5.7 min). Concentrations of 5–30 μg/mL of compound **1** were used. The calibration curve of **1** was determined by the linear equation: y = 29.222x + 7.5289 (R^2^ = 0.9952), where y is the area and x is the concentration (μg/mL). The content of compounds **2**, **3** and **4** was expressed as equivalents of compound **1**.

### 3.5. Cell lines and Cell Culture Conditions

Human foreskin fibroblasts (BJ) were obtained from the American Tissue Culture Collection (ATTC). BJ cells were maintained in Dulbecco’s modified Eagle’s medium (DMEM; ThermoFisher Scientific, Waltham, MA, USA), supplemented with 10% (*v*/*v*) fetal bovine serum (FBS), 2 mM glutamine and 1% non-essential amino acids [53]. Cells were maintained in a humidified environment of 5% CO_2_ and 37 °C. They were subcultured using a trypsin/EDTA solution (ThermoFisher Scientific, Waltham, MA, USA).

### 3.6. Measurement of Cathepsin B, L Enzymatic Activity

Cathepsin B, L activity was measured as described previously [54,55] with minor modifications. Human foreskin fibroblasts (BJ) were plated in 60-mm dishes and were treated for 24 h with a concentration of 1 μg/mL and 10 μg/mL of concentration for each extract and 5 μM for each compound. Cells were lysed on ice in 1 mM dithiothreitol and 50 mM Tris, pH 4.0 and the lysates were cleared at 14,000×*g* for 20 min at 4 °C. Following protein content measurement with Bradford assay (Bio-Rad laboratories, Hercules, CA, USA), 20 μg of protein were incubated in the reaction buffer (50 mM sodium acetate, 8 mM cysteine-hydrochloride, 1 mM EDTA, pH 4.0) containing the substrate z-FR-AMC (Enzo Life Sciences, Farmingdale, NY, USA) for 30 min at 37 °C. The fluorescence was measured (VersaFluorTM Fluorometer System, Bio-Rad laboratories) at excitation and emission wavelengths of 350 nm and 440 nm, respectively.

### 3.7. Proteasome Activity

Measurement of proteasome proteolytic activities was performed as previously described [22,56]. Briefly, human fibroblasts were plated in 60-mm dishes and were treated for 24 h with a concentration of 5 μM for each compound and 1 μg/mL and 10 μg/mL of concentration for each extract. After the treatment cells were lysed with a buffer suitable for the isolation of 26S proteasome (0.2% Nonidet P-40, 5 mM ATP, 10% glycerol, 20 mM KCl, 1 mM EDTA, 1 mM dithiothreitol and 20 mM Tris, pH 7.6). Lysates were cleared with centrifugation at 19,000g (4 °C) and 20 μg of proteins were immediately used to determine the main proteasome proteolytic activities [chymotrypsin-like (CT-L/LLVY) and caspase-like (C-L/LLE)]. Activities were assayed by recording the hydrolysis of the fluorogenic peptides Suc–Leu–Leu–Val–Tyr–AMC and Z-Leu–Leu–Glu–AMC (Enzo Life Sciences), at 37 °C for 30 min. The fluorescence was measured at a VersaFluorTM Fluorometer System (Bio-Rad laboratories) at excitation and emission wavelengths of 350 and 440 nm, respectively. Each sample was prepared in duplicate.

### 3.8. Cytotoxicity

Cytotoxicity was evaluated on A2058 and HepG2 cell lines by the ΜΤΤ method, as well as on CCD25sk cell line by the HOECHST assay, following a previously described process [57,58,59].

### 3.9. Molecular Simulations

Molecular Docking Simulations were run using the crystal structure of pro-cathepsin B (PDBid: 2PBH) and pro-cathepsin L (PDBid: 1CS8). Enzymes we prepared based on the Protein Preparation Wizard as implemented on Maestro 11.5 (Schrödinger, LLC, New York, NY, 2018). Induced fit algorithm was utilized for Molecular Docking as implemented on Maestro 11.5. The active site was defined using the coordinates of residues HisP^28^, LysP^39^ and ArgP^40^ which have been identified as crucial for GAG binding, by mutational analysis [50]. Ligands were designed using Maestro 11.5 and prepared by using the Ligand Preparation workflow as implemented on Maestro 11.5 (Schrödinger, LLC, NY, USA).

### 3.10. Statistical Analysis

Experiments were performed in duplicates or triplicates as indicated in Figure legends. For statistical analyses MS Excel and the Statistical Package for Social Sciences (IBM SPSS; version 19.0 for Windows, Armonk, NY, USA) were used. Statistical significance was evaluated using one-way analysis of variance (ANOVA). Data points correspond to the mean of the independent experiments and error bars denote standard deviation (SD).

## 4. Conclusions

Fungal extracts have been used for a long time as a source of antioxidant compounds, as well as for their ability to regulate the immune system, inhibiting tumor cell growth and exhibiting anti-aging effects [60]. Herein, we show that benzoic acid derivatives (**1**–**4**) isolated from *B. adusta* enhance the activity of the two main cell protein degradation systems, namely ALP and UPP (with the exception of compound **2** for UPP) and especially the activity of cathepsins B and L. Our in silico studies support the fact that all the isolated compounds are possible binders of both procathepsin B and L, and thus suggest a likely direct effect on cathepsins activity. Deregulation of both pathways (UPP and ALP) is often associated to aging and age-related diseases. Thus, the development of novel agents able to suppress the age-related decline of UPP and/or ALP activity could be considered as promising candidates for novel formulations with anti-aging properties.

## Figures and Tables

**Table 1:** Quantification results of compound **1**-**4** in EtOAc and EtOAc-LL extracts of CF-092983.

Compound	EtOAc LL Extract (μg mg^−1^)	EtOAc Extract (μg mg^−1^)
**1**	17.57 ± 0.18	2.68 ± 0.17
**2** *	2.46 ± 0.13	1.03 ± 0.05
**3** *	7.99 ± 0.08	1.39 ± 0.12
**4** *	4.43 ± 0.04	0.89 ± 0.03

* The content of compounds **2**, **3** and **4** was expressed as equivalents of compound **1**.

## Supplementary Materials

The following are available online, Figure S1: HPLC chromatogram of the Hex (A), EtOAc (B), EtOAc-LL (C) and MeOH Xad4 extracts of the strain CF-0902983 at 210 nm., Figure S2: ^1^H-NMR spectrum of 4-hydroxy-benzoic acid (**1**) in CD_3_OD, Figure S3: ESI(−)-HRMS spectrum of 4-hydroxy-benzoic acid (**1**), Figure S4: ^1^H-NMR spectrum of 4-methoxybenzoic acid (**2**) in CD_3_OD, Figure S5: ESI(+)-HRMS spectrum of 4-methoxybenzoic acid (**2**), Figure S6: ^1^H-NMR spectrum of 3-chloro-4-methoxy benzoic acid (**3**) in CD_3_OD, Figure S7: ESI(−)-HRMS spectrum of 3-chloro-4-methoxy benzoic acid (**3**), Figure S8: ^1^H-NMR spectrum of 3,5-dichloro-4-methoxybenzoic acid (**4**) in CD_3_OD, Figure S9: HSQC-NMR spectrum of 3,5-dichloro-4-methoxybenzoic acid (**4**) in CD_3_OD, Figure S10: HMBC NMR spectrum of 3,5-dichloro-4-methoxybenzoic acid (**4**) in CD_3_OD, Figure S11: ESI(−)-HRMS spectrum of 3,5-dichloro-4-methoxybenzoic acid (4); NMR data

## Abbreviations

ALP	autophagy-lysosome pathway
ATP	adenosine triphosphate
CAM	chlorinated anisyl metabolites
CH3CN	acetonitrile
C-L/LLE	caspase-like activity of the proteasome
CT-L/LLVY	chymotrypsin-like activity of the proteasome
DNA	deoxyribonucleic acid
EDTA	ethylenediaminetetraacetic acid
EtOAc	ethyl acetate
EtOH	ethanol
FBS	fetal bovine serum
GAGs	glycosaminoglycans
H_2_O	water
Hex	hexane
HPLC	high-performance liquid chromatography
UPLC-HRMS	ultra-high performance liquid chromatography coupled high resolution mass spectrometry
MeOH	methanol
NMR	nuclear magnetic resonance
PCR	polymerase chain reaction
PN	proteostasis network
TFA	trifluoroacetic acid
UPP	ubiquitin-proteasome pathway
