# Supplementary material for: Biological Evaluation and In Silico Study of Benzoic Acid Derivatives from Bjerkandera adusta Targeting Proteostasis Network Modules
Source: Molecules. 2020 Feb 4;25(3):666. doi: 10.3390/molecules25030666 (PMC7036779; doi:10.3390/molecules25030666)
Supplement: Supplementary file 1 [file molecules-25-00666-s001.pdf]

SUPPLEMENTARY MATERIAL

Biological Evaluation and In Silico Study of Benzoic Acid Derivatives from *Bjerkandera adusta* Targeting Proteostasis Network Modules

Katerina Georgousaki [1], Nikolaos Tsafantakis [1], Sentiljana Gumeni [2], George Lambrinidis [3], Victor González-Menéndez [4], Jose R. Tormo [4], Olga Genilloud [4], Ioannis P. Trougakos [2] and Nikolas Fokialakis [1,*]

[1] Division of Pharmacognosy and Natural Products Chemistry, Department of Pharmacy, National and Kapodistrian University of Athens, Athens 157 71 Greece; kat_georgousaki@hotmail.com (K.G.); ntsafantakis@pharm.uoa.gr (N.T.)

[2] Department of Cell Biology and Biophysics, Faculty of Biology, National and Kapodistrian University of Athens, Athens, 157 72 Greece; sgumeni@biol.uoa.gr (S.G.); itrougakos@biol.uoa.gr (I.P.T.)

[3] Division of Pharmaceutical Chemistry, Department of Pharmacy, National and Kapodistrian University of Athens, Athens, 157 84 Greece; lamprinidis@pharm.uoa.gr

[4] Fundacion MEDINA, Health Sciences Technology Park, Granada 18016, Spain; victor.gonzalez@medinaandalucia.es (V.G.-M.); ruben.tormo@medinaandalucia.es (J.R.T.); olga.genilloud@medinaandalucia.es (O.G.)

* Correspondence: fokialakis@pharm.uoa.gr; (N.F.) Tel.:++30 210 727 4727

[*] Author of correspondence:

Dr. Nikolas Fokialakis: fokialakis@pharm.uoa.gr

List of Supplementary Material

Figure S1: HPLC chromatogram of the Hex (A), EtOAc (B), EtOAc L-L (C) and MeOH Xad4 extracts of the strain CF-0902983 at 210 nm. 3

Figure S2. ^{1}H NMR spectrum of 4-hydroxy-benzoic acid (**1**) in CD_3OD 4

Figure S3. ESI(-)-HRMS spectrum of 4-hydroxy-benzoic acid (**1**) 4

Figure S4. ^{1}H NMR spectrum of 4-methoxybenzoic acid (**2**) in CD_3OD 5

Figure S5. ESI(+)-HRMS spectrum of 4-methoxybenzoic acid (**2**)........ 6

Figure S6. ^{1}H NMR spectrum of 3-chloro-4-methoxy benzoic acid (**3**) in CD_3OD........ 7

Figure S7. ESI(-)-HRMS spectrum of 3-chloro-4-methoxy benzoic acid (**3**)........ 7

Figure S8. ^{1}H NMR spectrum of 3,5-dichloro-4-methoxybenzoic acid (**4**) in CD_3OD 8

Figure S9. HSQC NMR spectrum of 3,5-dichloro-4-methoxybenzoic acid (**4**) in CD_3OD 8

Figure S10. HMBC NMR spectrum of 3,5-dichloro-4-methoxybenzoic acid (**4**) in CD_3OD 9

Figure S11. ESI(-)-HRMS spectrum of 3,5-dichloro-4-methoxybenzoic acid (**4**) 9

NMR data 10

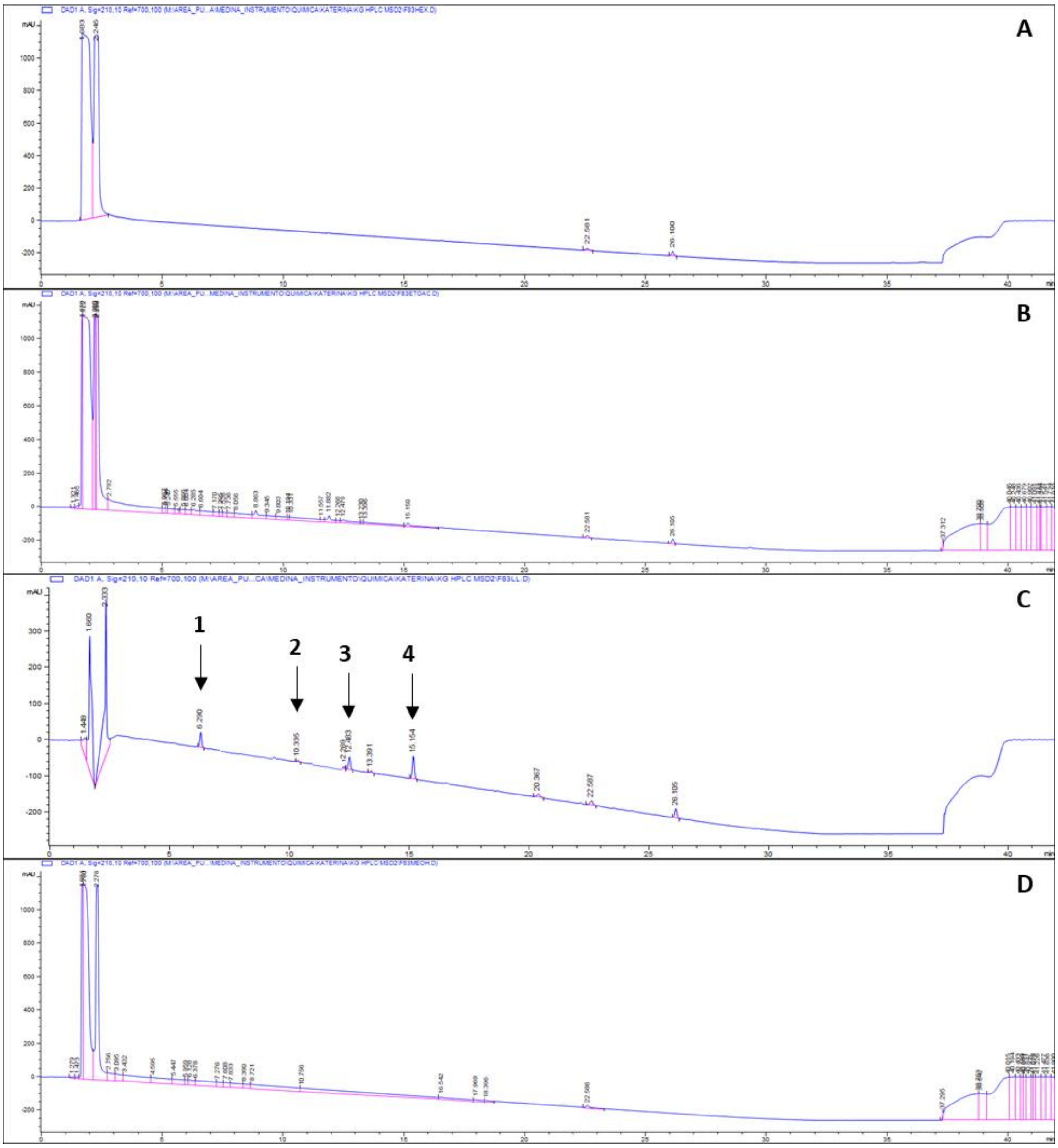

Figure S1: HPLC chromatogram of the Hex (A), EtOAc (B), EtOAc L-L (C) and MeOH Xad4 extracts of the strain CF-0902983 at 210 nm.

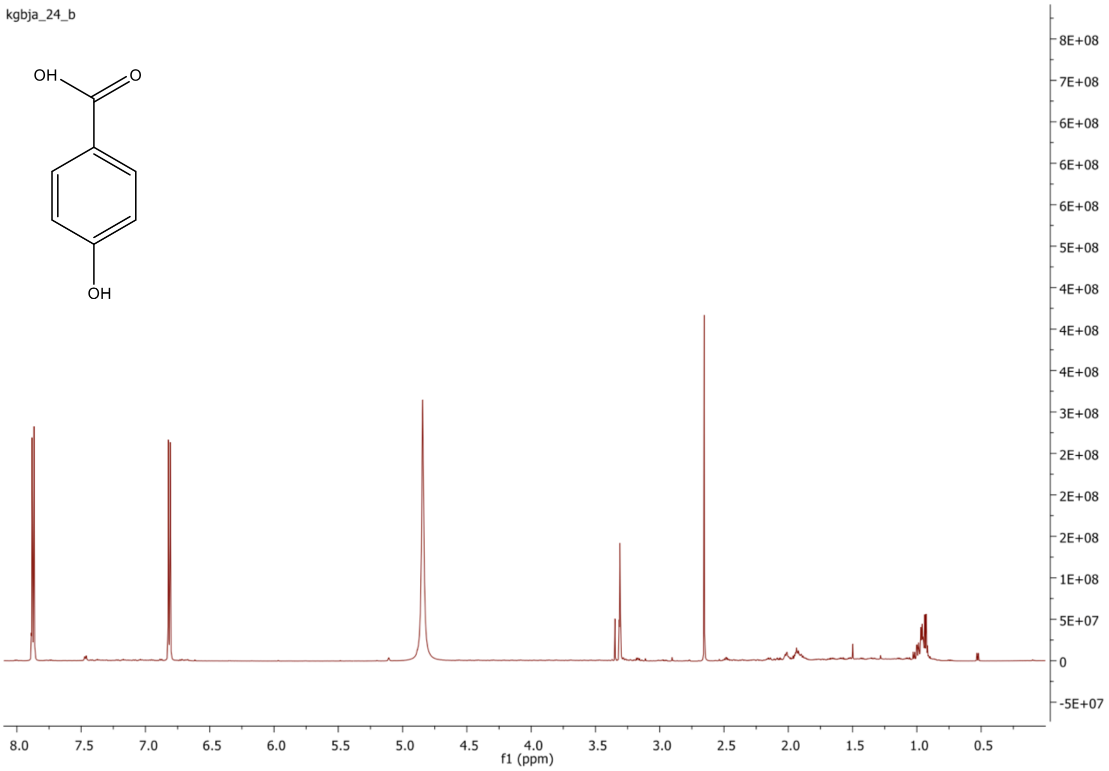

Figure S2. ^{1}H NMR spectrum of 4-hydroxy-benzoic acid (**1**) in CD_3OD

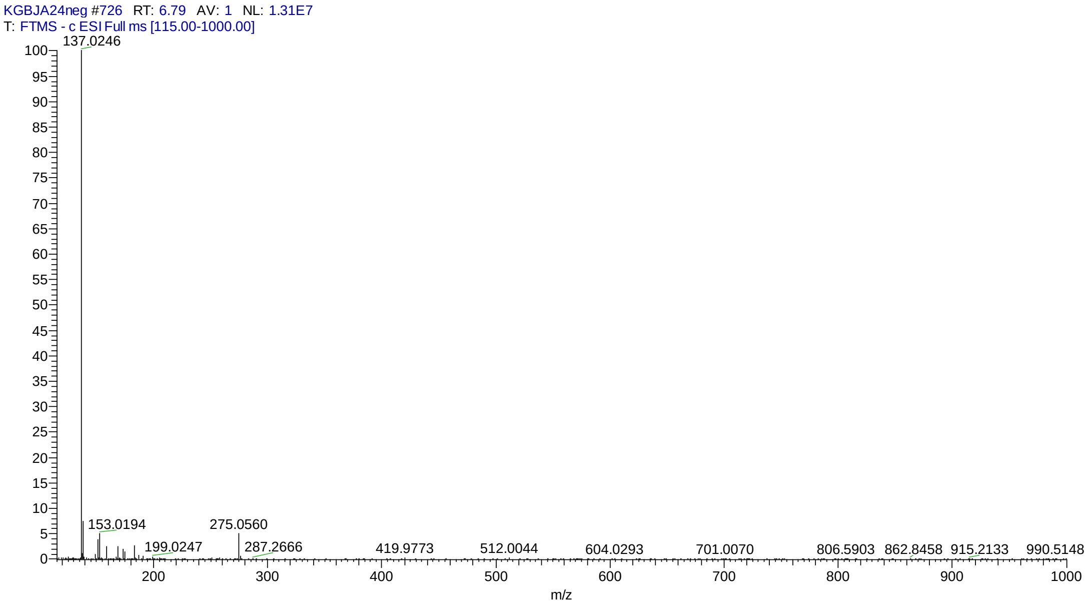

Figure S3. ESI(-)-HRMS spectrum of 4-hydroxy-benzoic acid (**1**)

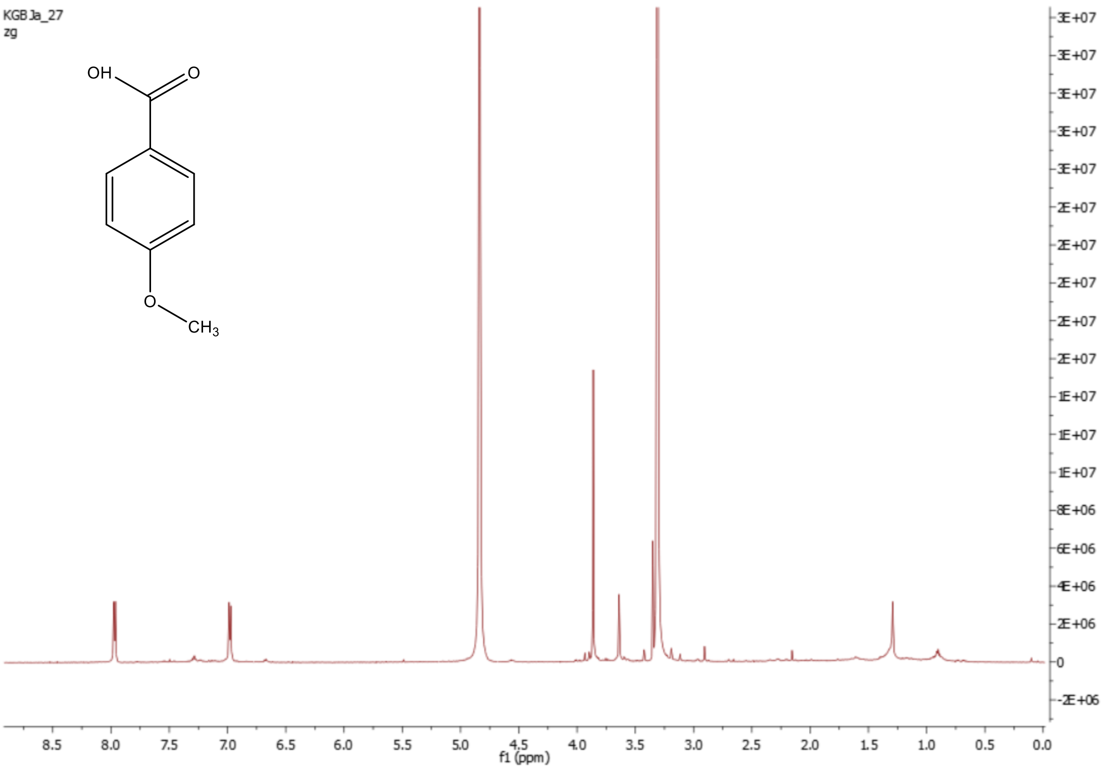

Figure S4. ^{1}H NMR spectrum of 4-methoxybenzoic acid (**2**) in CD_3OD

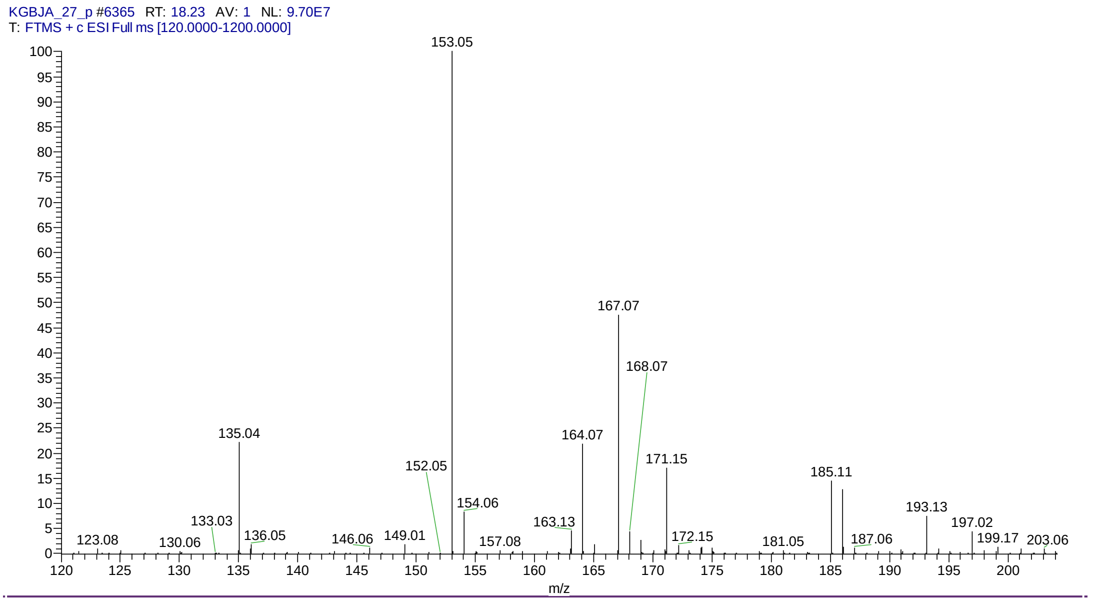

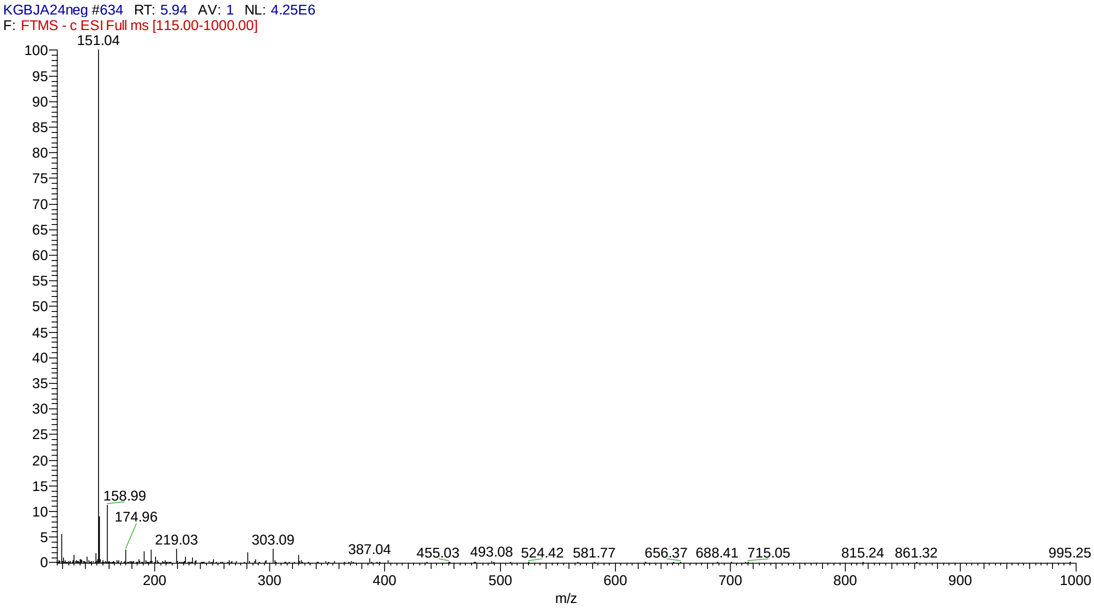

Figure S5. ESI~~(+)~~ (-)-HRMS spectrum of 4-methoxybenzoic acid (**2**)

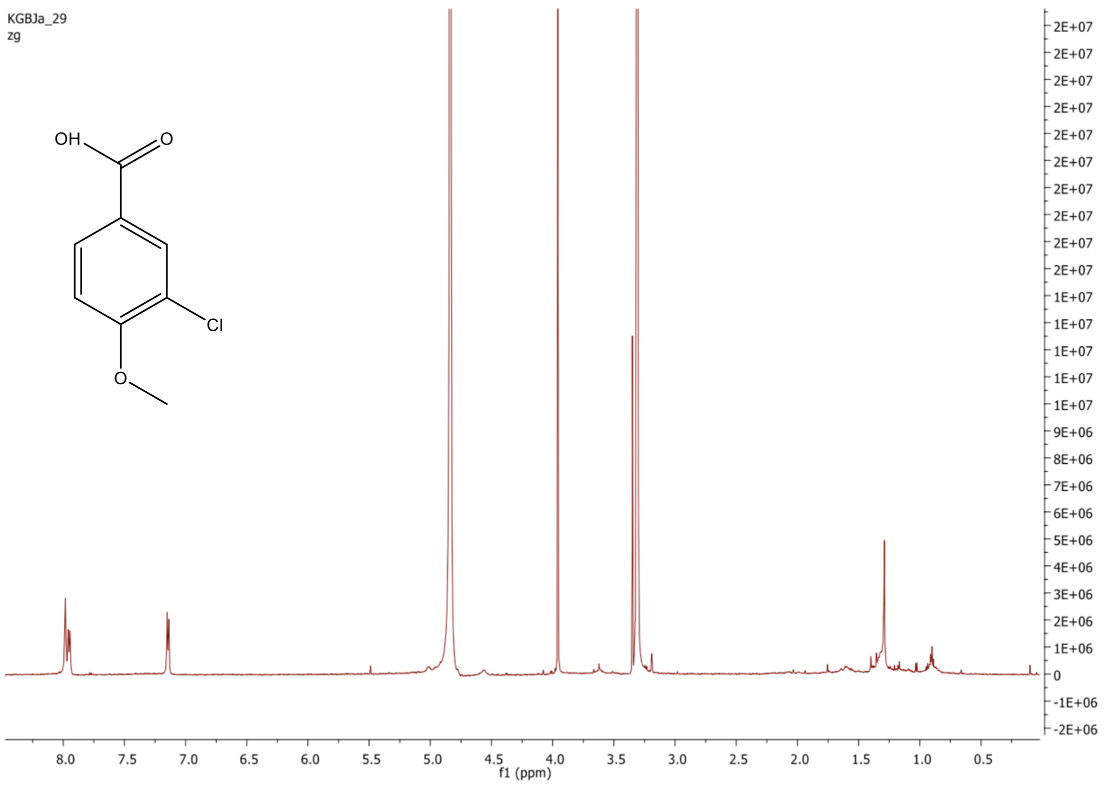

Figure S6. ^{1}H NMR spectrum of 3-chloro-4-methoxy benzoic acid (**3**) in CD_3OD

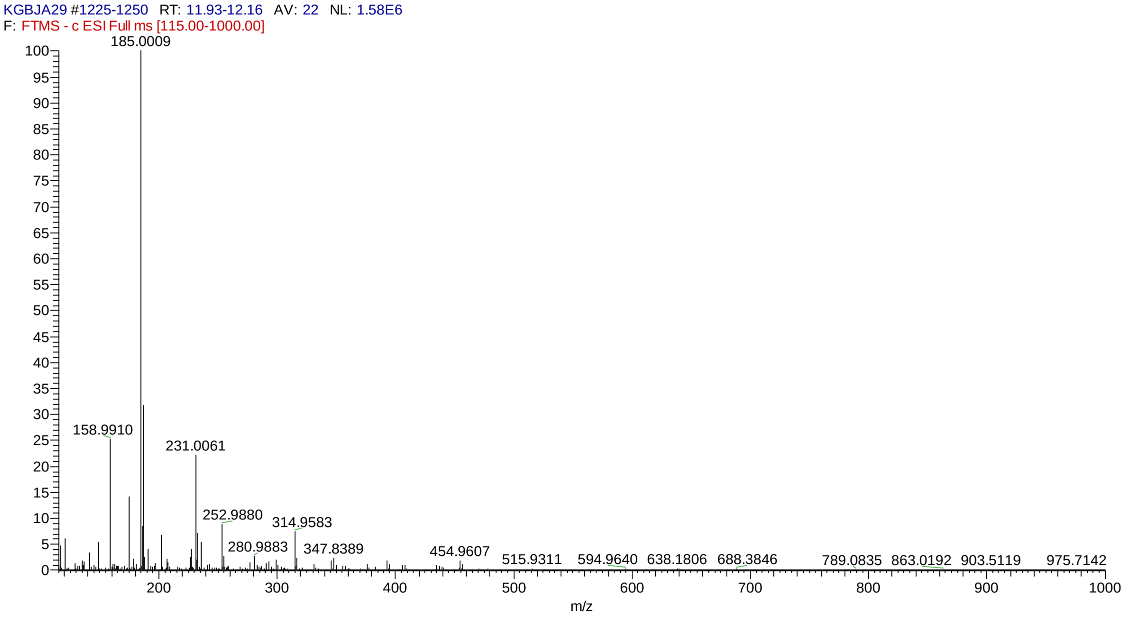

Figure S7. ESI(-)-HRMS spectrum of 3-chloro-4-methoxy benzoic acid (**3**)

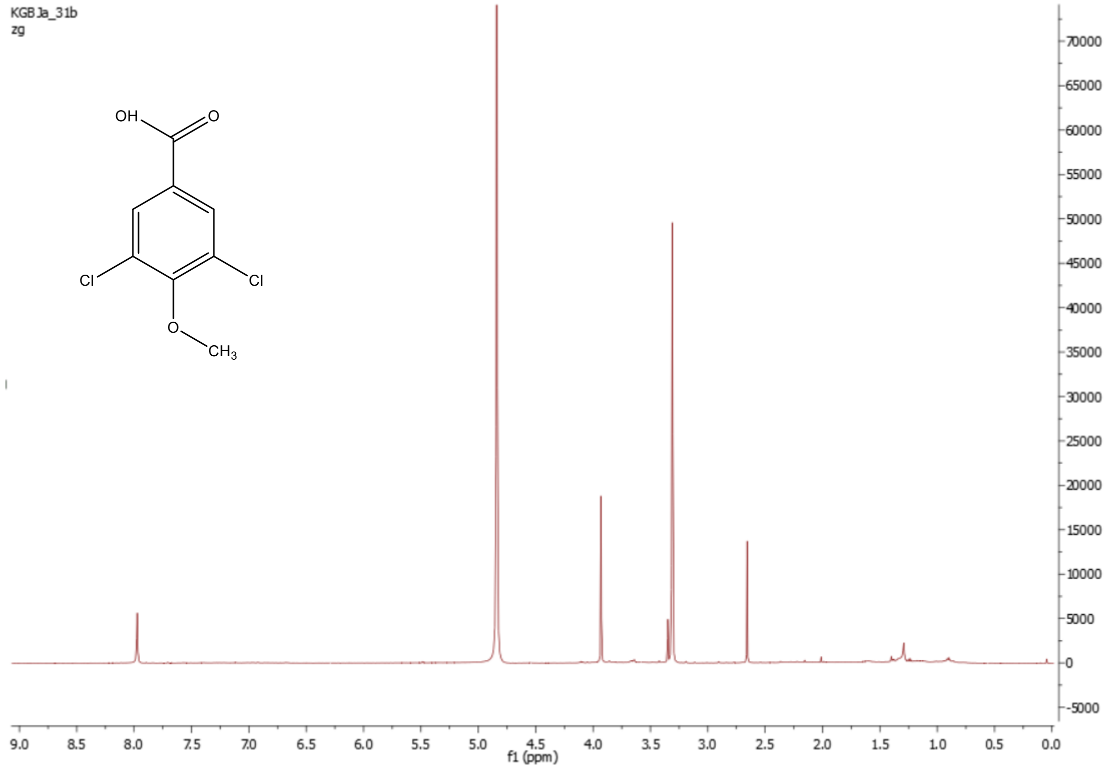

Figure S8. ^{1}H NMR spectrum of 3,5-dichloro-4-methoxybenzoic acid (**4**) in CD_3OD

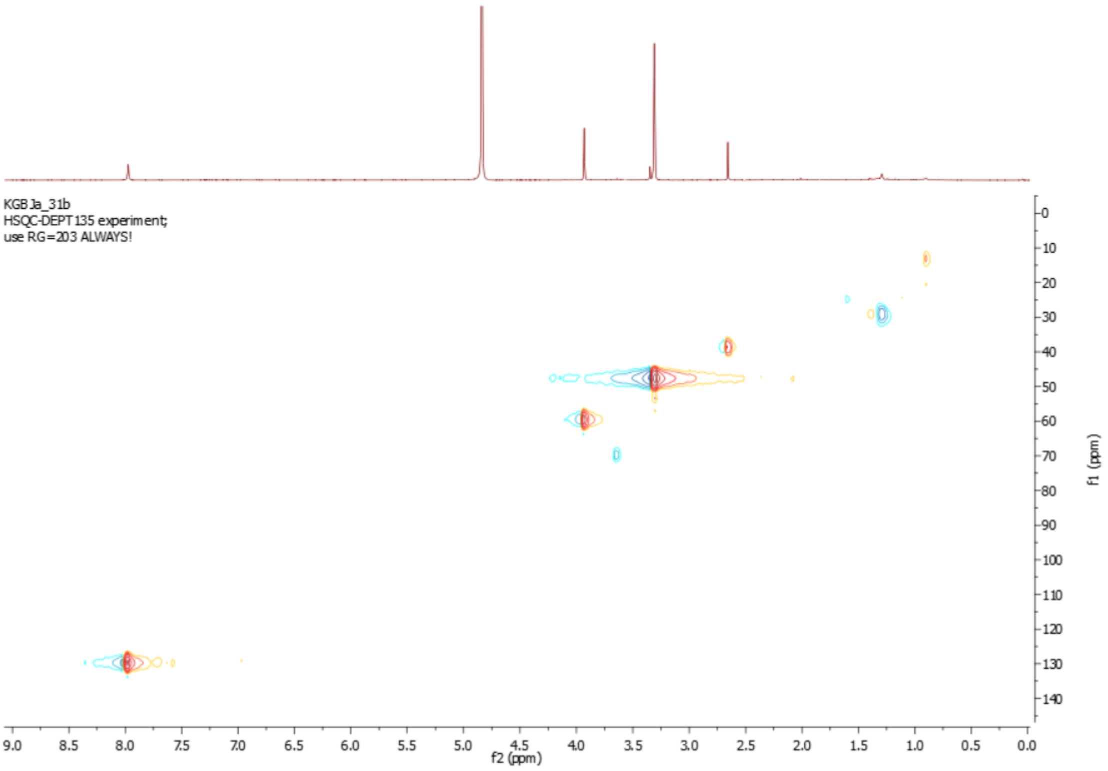

Figure S9. HSQC NMR spectrum of 3,5-dichloro-4-methoxybenzoic acid (**4**) in CD_3OD

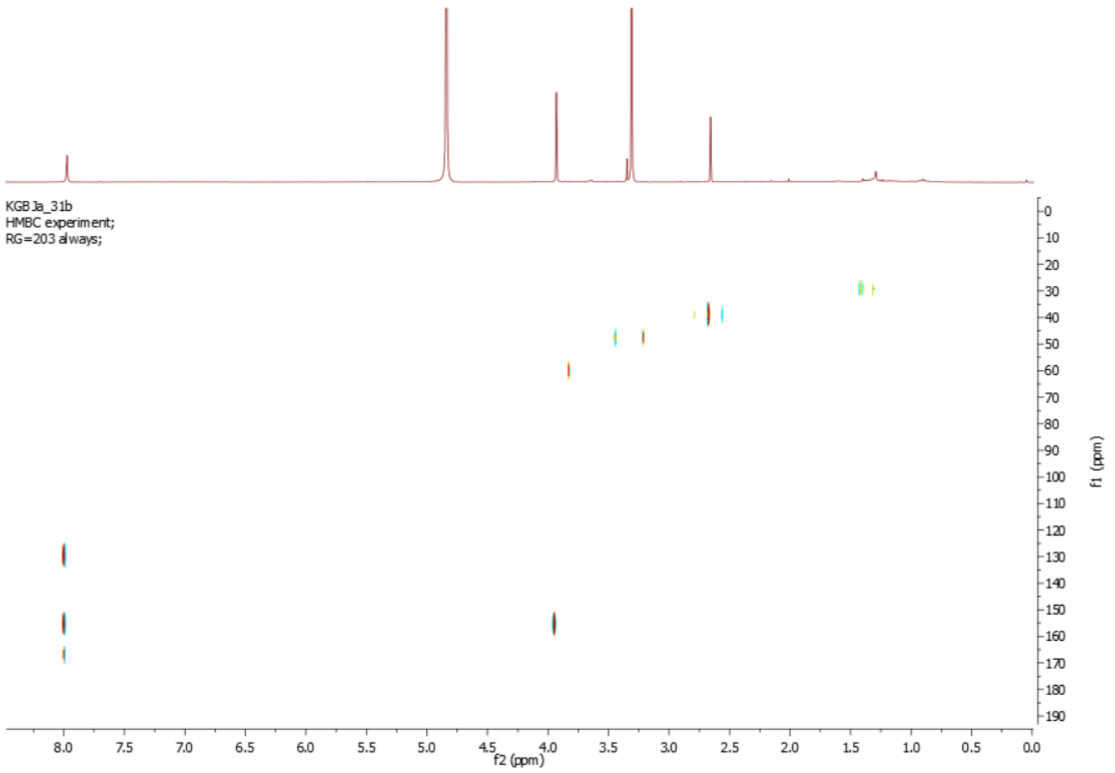

Figure S10. HMBC NMR spectrum of 3,5-dichloro-4-methoxybenzoic acid (**4**) in CD_3OD

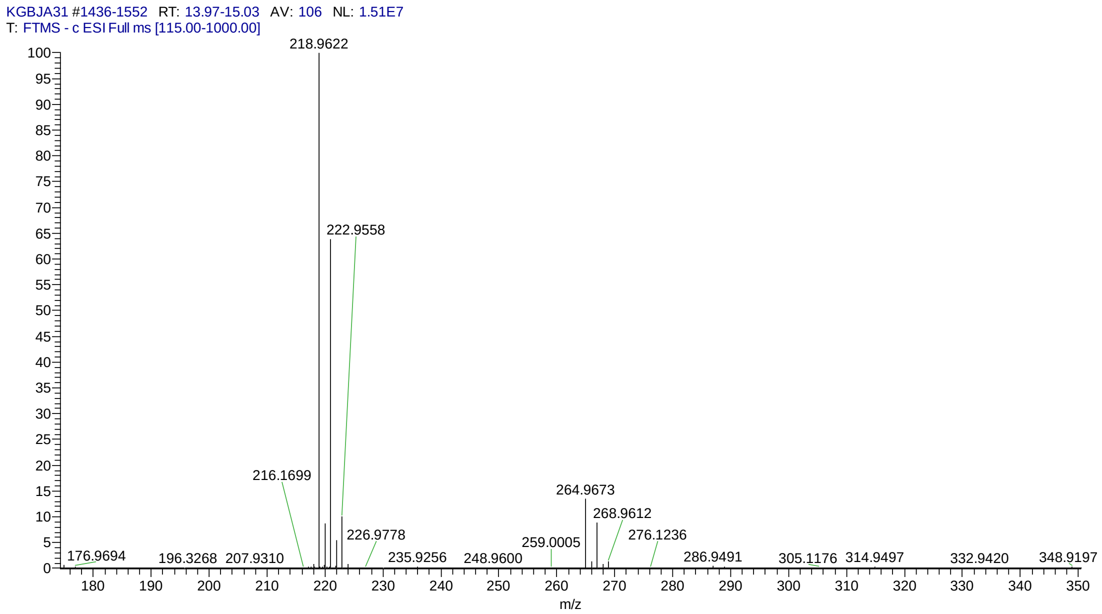

Figure S11. ESI(-)-HRMS spectrum of 3,5-dichloro-4-methoxybenzoic acid (**4**)

NMR data

4-hydroxy-benzoic acid (**1**) Yellow-brown solid; UV (MeOH) λmax: 208, 256 nm; ^{1}H NMR (CD_3OD, 600 MHz): 7.87 (d, J=8.68 Hz, H-2/H-5), 6.81 (d, J=8.78 Hz, H-3/H-6; ^{13}C NMR (CD_3OD, 600 MHz); ESIMS negative *m/z* $[M-H]^-$ 137.0246 (calcd for $C_7H_6O_3$, 138.12074).

4-methoxy-benzoic acid (**2**) white solid; UV (MeOH) λmax: 200, 256 nm; ^{1}H NMR (CD_3OD, 600 MHz): 7.97 (d, J=8.77 Hz, H-2/H-5), 6.98 (d, J=8.71 Hz, H-3/H-6; ESIMS positive *m/z* $[M-H]^+$ 153.0544 (calcd for $C_8H_8O_3$, 152.14732).

3-chloro-4-methoxy benzoic acid (**3**) yellow solid; UV (MeOH) λmax: 200, 256 nm; ^{1}H NMR (CD_3OD, 600 MHz): 7.94-8.00 (m, H-2/H-5), 7.14 (d, J=8.21 Hz, H-3/H-6), 3.96 (s, 4-OCH_3); ESIMS negative *m/z* $[M-H]^-$ 185.0009, (calcd for $C_8H_7ClO_3$, 186.59238).

3,5-dichloro-4-methoxybenzoic acid (**4**) yellow solid; UV (MeOH) λmax: 200, 243 nm; ^{1}H NMR (CD_3OD, 600 MHz): 7.97 (brs, H-2/H-6), 3.93 (s, 4-OCH_3); ESIMS negative *m/z* $[M-2H]^-$ 218.9622, (calcd for $C_8H_6Cl_2O_3$, 221.03744).
